# Comparative study of conventional versus digital bitewing radiographs for estimating alveolar bone level

**DOI:** 10.6026/973206300213147

**Published:** 2025-09-30

**Authors:** Trishna Saikia, Polysmita Ojah, Himanshi Tomar, Queentaj Dutta, Dharitri Bharali, Murchana Mahanta

**Affiliations:** 1 Department of Oral medicine and Radiology, Government Dental College, Dibrugarh, Assam, India; 2 Department of Prosthodontics and Crown and Bridge, Government Dental College, Dibrugarh, Assam, India; 3 Department of Oral medicine and Radiology, IDST Ghaziabad, Uttar Pradesh, India; 4 Department of Conservative Endodontic and Aesthetic Dentistry, Government Dental College, Dibrugarh, Assam, India; 5 Department of Public Health Dentistry, Government Dental College, Dibrugarh, Assam, India; 6 Department of Dentistry, Government Dental College, Dibrugarh, Assam, India

**Keywords:** Alveolar bone level (ABL), Conventional radiography, Cemento enamel junction (CEJ), Digital radiography (RVG), Periodontitis, Paired t-test

## Abstract

Conventional and digital bitewing radiographs were taken for right posterior segments and analysis was done. Hence, 50 patients comprising of both male and females aged between 20-70 years were randomly selected for the study. The values obtained from two independent radiologists in conventional bitewing radiographs and with SOPRO imaging digital software in digital bitewing radiographic methods were compared to estimate the amount of bone height obtained through each method. No significant difference was found between the mean values of alveolar bone level measured by the two types of radiographs for all the sites; and is almost similar to those revealed on conventional radiographs.Thus, we show either of the two radiographic techniques *i.e.*, conventional and digital bitewing radiographs can be used interchangeably in epidemiologic studies.

## Background:

Diagnosis is the process of evaluation of a patient's health as well as deriving opinions formulated by clinicians. Oral diagnosis is the technique of utilizing scientific knowledge to ascertain the nature of oral diseases and in differentiating it from other diseases [1, 2]. Radiographs play an essential part in diagnostic dentistry. Many pathologic or abnormal conditions and their presence and extension can be traced by means of radiography. Radiography is a well-established procedure in daily dental practice and is still the most basic and an important available diagnostic tool. Radiographs play a fundamental role in the assessment of periodontal diseases. Conventional bitewing and intra oral periapical radiographs are frequently used to detect alveolar bone loss associated with periodontal disease. They provide unique details about the status of the periodontium and a permanent record of the bone throughout the course of the disease. However, the quality of an X-ray sensitive film can be affected by multiple variables such as improper exposure, under or overdeveloping and poor fixing [3]. Over the past few years, systems that can generate radiographic digital images without the need for radiographic film have become available for use in clinical practice and are gaining popularity among practitioners. Such digital radiography can also reduce the radiation exposure. One of the most useful advantages of digital radiography is the knack it provides to the clinicians to send images to practitioners 2 in a matter of minutes, for which it has become widely accepted as an alternative to film-based radiography [1, 2]. Periodontal disease is one amongst the most commonly occurring diseases in the general population. Evidence suggests that periodontal bone loss could commence at an earlier age than previously thought [4, 5]. Plaque related gingival inflammation is associated with the release of numerous inflammatory mediators such as interleukin 1(IL-1), tumor necrosis factor - alpha (TNF α) and prostaglandin - E2 (PGE-2) 2,6. The generated inflammatory mediators reach the alveolar housing of the tooth and disrupt the balance between bone resorption and bone apposition, resulting in the loss of alveolar bone [6, 7 and 8]. The detection of diseases of periodontium often depends on conventional bitewing radiographs. The distortion with bitewing radiographs are relatively minimal, but their diagnostic accuracy is however notoriously imprecise [9, 10]. These diagnostic challenges are well targeted by technological initiative that appears to lie in the existing scientific capabilities. Even more tantalizing is the possibility of exploiting the new digital image technology to facilitate characterization of relatively small site-specific changes [11, 12]. Alveolar bone height is the distance between the cemento enamel junction (CEJ) and the alveolar bone crest (ABC), along a line parallel to the long axis of the tooth [13]. This distance indicates whether there is alveolar bone loss (ABL) and bone alterations due to periodontal disease. Although studies showed considerable variation in this distance, from 0 to 3 mm, a 2 mm distance is frequently adopted as the standard for patients without periodontal disease. The diagnosis of periodontal diseases requires a careful clinical examination that takes into account the clinical history and associated signs and symptoms. In addition, 3 radiographic examinations is a fundamental tool to assess morphological and pathological changes in the periodontium, to assist the diagnosis, treatment planning and prognosis of periodontal diseases [14]. The use of radiographs as a diagnostic tool has become an indispensable routine in medicine and dentistry. The presence and extension of many pathologic or abnormal conditions can be traced by means of radiographs. A recent advance in computer technology has led to the development of digital imaging, which has made a significant impact on dental radiographs [15]. Direct digital imaging uses a digital sensor or detector in place of film for capturing images. On exposure to X-rays, an electronic image accumulates as a pattern of charge on the detector array. Digital imaging offers some distinct advantages over film in terms of exposure reduction, elimination of processing chemicals, instant or real time image production, image enhancement, patient education utility and convenient storage. Therefore, it is of interest to show in recent years, a digital imaging system - Radiovisiography (RVG) has provided an alternative and instant method for measurement of intraoral radiography. It has been reported that RVG system provided approximately an 80% reduction in radiation dosage in comparison with conventional X-ray films [16]. Therefore, it is of interest to compare conventional vs. digital bitewing radiographs for estimating alveolar bone level.

## Materials and Methods:

50 patients were randomly selected from Out-patients consulting the Department of Oral Medicine, Diagnosis and Radiology, out of which 34 males and 16 females was included in the study to compare the alveolar bone level through conventional and digital bitewing radiographs.

## Inclusion criteria:

[1] Patients aged between 20-70 years.

[2] Patients with horizontal bone loss.

[3] Ideal bitewing radiographs of patients.

## Exclusion criteria:

[1] Patients with drifted, supernumerary, crowded, interproximal caries.

[2] Overhanging restorations, wearing intraoral appliance and missing posteriors.

[3] Pregnant patients.

[4] Patients contraindicated for any radiographic procedure.

## Procedure:

The patients were explained for the need and design of the study and approved consent was obtained from them. The patient's demographic data and general history was then entered in a predesigned proforma. Oral examination was carried out and a relevant finding was entered in the proforma. Each of the patients was asked to wear lead apron and thyroid shield and then a set of conventional bitewing radiographs was taken for right posterior segments using RinnXcp (red) posterior bitewing holders and Carestream E-speed film of size 30.5 x 40.5 mm using Satelec dental x-ray unit operated at 70 Kvp, 8mA and exposure time of 0.4-0.5 seconds. The exposed film was developed using time and temperature method. Then another set of digital radiographs was taken for the above mentioned teeth at the same time using RVG with Sopix2 sensor (size1) of 20x30 mm at 70 Kvp, 8mA and exposure time of 0.12- 0.2 seconds using.

## Sopix2 ace RVG unit:

The conventional radiographic images were assessed by two independent oral radiologists. The conventional bitewing radiographs were then be mounted on a x-ray viewer, keeping CEJ as the reference point, alveolar bone height was measured by placing divider on the CEJ to the crest of the alveolar bone. Later transparent ruler was used to evaluate the distance between the two points of the divider. Measurement in RVG was done by using Soproimaging digital software. The values obtained from two independent radiologists through conventional and via sopro imaging digital software in digital bitewing radiographic methods was compared to estimate the amount of bone height obtained through each method.

## Method of collection of data:

Convenient sampling method will be used to select the sample group as per the inclusion and exclusion criteria after obtaining informed consent from the patient.

## Results:

Study population (n=50) composed of randomly selected male and female patients aged between 20-70 years were selected out of which 34 were males and 16 were females. Two examiners independently performed the radiographic measurements of alveolar bone levels in conventional radiographs. Sopro imaging digital software was used to do the radiographic measurement in digital radiographs. For analysis, these observations were averaged over distal and mesial aspects of 1st molar and 2nd molar between examiner 1 and 2 and between conventional and digital radiographs as shown in Table 1 (see PDF).

As shown in Table 2 (see PDF), Comparison of mean values of alveolar bone level measured by Conventional bitewing radiographs between the 2 Radiologists (examiners) using paired t-test in the 1st molar (46)-Examiner 1 measured a mean bone loss of 2.20 mm ± 0.881 in the distal aspect of 1st molar while examiner 2 measured an average bone loss of 2.24 mm ± 1.287, in the distal aspect of 1st molar which was not statistically significant. In the mesial aspect of 1st molar, a mean bone loss of 2.52 mm ± 1.594 and 2.18 mm ± 1.815 was recorded by examiner 1 examiner 2 respectively and which was not statistically significant. Comparison of mean values of alveolar bone level measured by conventional bitewing radiographs between the 2 Radiologists (examiners) using paired t-test in the 2nd molar (47). Examiner1measuredamean bone loss of 2.28 mm ± 1.852 in the distal aspect of 2ndmolar while examiner 2 measured an average bone loss of 1.86 mm ± 2.070, in the distal aspect of 2nd molar, which was statistically not significant. In the mesial aspect of 2nd molar, a mean bone loss of 1.46 mm ± 1.515 was recorded by examiner1 whereas; examiner 2 measured a mean bone loss of 1.24 mm ± 1.598 and was not statistically significant.

[1] In Table 3 (see PDF), a strong positive correlation was obtained when comparison was done between digital and conventional methods for evaluating alveolar bone level in the distal and mesial aspects of 1st molar with a value of r= 0.625 andr= 0.650 respectively.

[2] Similarly, regarding the 2nd molar a strong positive correlation was obtained for alveolar bone level measurement in the distal aspect with a value of r= 0.646 and a very strong positive correlation was obtained in the mesial aspect with a value of r= 0.710.

[3] Highly significant correlation was found between conventional and digital radiograph for alveolar bone level measurements (p<0.001).

## Discussion:

Radiographs provide unique information about the status of the periodontium and a permanent record of the condition of the bone throughout the course of the disease and treatment. Radiographs help the clinician in identifying the extent of destruction of alveolar bone, local contributing factors and features of the periodontium that influence the prognosis [3]. The diagnosis of periodontal disease is primarily based on clinical examination and confirmed by radiographic examination, but the radiographs on its own cannot help in diagnosing the disease [17]. Khocht *et al.* 2003 stated that digital radiography offers many advantages over conventional methods [2]. The clinical implications of radiography in the diagnosis of periodontal disease are twofold; to visualize the initial status of the bone tissue and to illustrate changes in bone tissue over time. When there are so many radiographic techniques, the clinician is in a dilemma as to which technique has to be used. This study was an attempt to help the clinician select the reliable radiographic method in imaging and detection of periodontal osseous destruction [18]. It eliminates the need for film and film developing and has lower radiation exposure. The generated image is available immediately for evaluation on a computer screen and can be manipulated digitally to enhance viewing. In addition, digital tools are available to record electronic measurements and to cut, paste and colorize the image. The image can be easily filed on and retrieved from the hard disk or removable storage medium, or the images can be transferred electronically to third party carriers [19]. Engebretson *et al.* stated that there is no significant difference in conventional and digital bitewing radiographs as such but digital radiographs are more accurate in measurements than film- based radiographs [20].

A study done by Singh *et al.* 2015 it was observed that in the normal clinical use, significant difference exists between alveolar bone loss measurements on digital and conventional radiographs in several regions in the mouth [1]. This was contrary to our study, which showed similar results by both digital and conventional bitewing radiographs. In a study conducted by Ahmed Khocht *et al.* 2003 digital radiography measured 0.3 mm greater bone loss than conventional bitewings with significant p-value and digital radiographs observed bone loss only in the posterior mandibular region and measurements of bone loss in the posterior maxillary region were similar between the two radiographic methods [2]. This was contrary to our study which showed negligible difference in the measurements between conventional and digital bitewing methods with statistically insignificant P-value in all the sites undertaken for the study. From our study, no significant difference between mean alveolar bone level measurements in the two types of radiographs for all the sites was found. We can conclude that either of the two radiographic techniques may be used interchangeably in epidemiologic studies. Even though both are useful in detecting the interdental bone level, digital bitewing radiographs is superior to conventional radiographs for the detection of interdental bone level due to reduced time and radiation exposure to obtain the same diagnostic information but cost can be a negative factor. Hence, further studies can be performed with a larger sample size, with all the quadrants of the maxilla and mandible and with active periodontitis before and after treatment to conclude the results from both the methods.

## Conclusion:

No significant difference was observed in mean alveolar bone level measurements between conventional and digital bitewing radiographs. Both methods can be used interchangeably in epidemiological studies, though digital radiographs offer advantages such as reduced time and radiation exposure. Further studies with larger sample sizes and broader site inclusion are recommended to validate these findings.
